# Compound heterozygous mutations in *FBN1* in a large family with Marfan syndrome

**DOI:** 10.1002/mgg3.1116

**Published:** 2020-01-16

**Authors:** Aideen M. McInerney‐Leo, Jennifer West, Lawrie Wheeler, Paul J. Leo, Kim M. Summers, Lisa Anderson, Matthew A. Brown, Malcolm West, Emma L. Duncan

**Affiliations:** ^1^ Dermatology Research Centre The University of Queensland Diamantina Institute University of Queensland Brisbane QLD Australia; ^2^ Translational Genomics Group Institute of Health and Biomedical Innovation Queensland University of Technology at Translational Research Institute Woolloongabba QLD Australia; ^3^ School of Clinical Medicine Prince Charles Hospital Clinical Unit The University of Queensland Brisbane QLD Australia; ^4^ Mater Research Institute‐University of Queensland Translational Research Institute Woolloongabba QLD Australia; ^5^ Department of Endocrinology Royal Brisbane and Women's Hospital Herston QLD Australia

**Keywords:** compound heterozygous, *FBN1*, *fibrillin 1*, Marfan syndrome

## Abstract

**Background:**

Marfan syndrome (MFS) is a dominant monogenic disorder caused by mutations in *fibrillin 1* (*FBN1*). Rarely, compound heterozygosity for *FBN1* mutations has been described.

**Methods:**

A large kindred with MFS was assessed clinically over decades, and genetically using exome and/or Sanger sequencing.

**Results:**

A previously identified *FBN1* missense variant (p.Tyr754Cys) was confirmed in all subjects with MFS. An additional variant (p.Met2273Thr), previously associated with incomplete MFS, was identified in three siblings. These three compound heterozygous individuals had aortic dilatation at early age (all <30 years): one also had cerebral and ocular aneurysms; and one, who had undergone surgical repair aged 18 years, died from aortic dissection at 31 years. In contrast, their heterozygous father (p.Tyr754Cys) with MFS died at 57 years (myocardial infarction) without requiring surgical intervention and one heterozygous (p.Tyr754Cys) sibling has aortic dilatation presenting >40 years but not requiring surgical intervention. Another heterozygous (p.Tyr754Cys) sibling did require aortic root repair (28 years). The heterozygous (p.Met2273Thr) mother had aortic dilatation diagnosed at age 68 years but has not required surgical repair.

**Conclusion:**

Although compound heterozygosity or homozygosity is rare in MFS, it should be considered when there is an unusually severe phenotype in a subset of family members.

## Graphical abstract

Marfan syndrome (MFS) is a dominant monogenic disorder caused by mutations in FBN1. Compound heterozygous mutations in FBN1 were identified in a large family with Marfan syndrome. Although compound heterozygosity or homozygosity is rare in MFS, it should be considered when there is an unusually severe phenotype in a subset of family members.

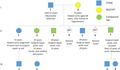

## 1 INTRODUCTION

Marfan syndrome (MFS; MIM 154700) is an autosomal dominant connective tissue disorder causing skeletal, ocular, and cardiovascular abnormalities (Marfan, 1896). Diagnosis of MFS is now standardized through the revised Ghent criteria (Loeys et al., 2010); using these criteria, the prevalence of MFS is 0.02%–0.03% (Judge & Dietz, 2005). MFS is caused by mutations in *fibrillin 1* (*FBN1;* MIM 134797) (Lee et al., 1991), identified in 90%–95% of individuals who meet the revised Ghent criteria (acknowledging that a *FBN1* mutation is among those criteria) (Baetens et al., 2011; Loeys et al., 2004, 2010; Robinson et al., 2006). There are some genotype–phenotype correlations due to the location and/or nature of the mutation within *FBN1*—for example, individuals with MFS and aortic involvement are much more likely to have truncating or splice site variants than individuals without aortic involvement (79% vs. 48%) (Baudhuin, Kotzer, & Lagerstedt, 2015); loss of function mutations within exons 24–32 are associated with neonatal and severe adult forms of MFS; and individuals with ectopia lentis are more likely to have a missense mutation affecting a cysteine residue than those individuals without (71% vs. 52%) (Faivre et al., 2007; Rommel et al., 2005). However, these correlations are modest; and overall, there is marked phenotypic variability between individuals with the same mutation, both inter‐ and intrafamilially (Dietz & Pyeritz, 1995; Dietz et al., 1992).

Here we report a large family with MFS and variably severe phenotype, at least part of which may be due to compound heterozygosity for *FBN1* mutations.

## 2 MATERIALS AND METHODS

### 2.1 Ethical clearance

This study was approved by The University of Queensland Human Research Ethics Committee (UQ #2011000876). All individuals provided written informed consent.

### 2.2 Participants

Members of a large family with MFS were recruited to a study using massive parallel sequencing in genetic disorders associated with bone dysplasias. The pedigree of this family has been previously published (Summers et al., 2004). Detailed clinical information was obtained from history, physical examination (including ophthalmology assessment), echocardiogram, and medical records, with all individuals with MFS evaluated every 3–4 years, over decades.

### 2.3 Whole‐exome sequencing

DNA was extracted from saliva or blood. A subset of family members from an extended pedigree (Summers et al., 2004) underwent whole‐exome sequencing (WES) to identify variants in MFS/thoracic aortic aneurysm genes, which could be modifying the severity. Libraries were prepared, exomes enriched, and sequence data were de‐multiplexed, processed, aligned, and annotated as described previously (McInerney‐Leo et al., 2016). Good quality, rare (minor allele frequency (MAF) <0.05) splice site, and coding variants in *FBN1, ACTA2, FOXE3, LOX, MAT2A, MFAP5, MYH11, MYLK, NOTCH1, PRKG1, SMAD3, TGFB2, TGFB3, TGFBR1,* and *TGFBR2* (Milewicz & Regalado, 2017) were assessed. Sanger sequencing was used to verify variants of interest and determine genotypes in pedigree members not undergoing WES.

## 3 RESULTS

**Figure 1:**
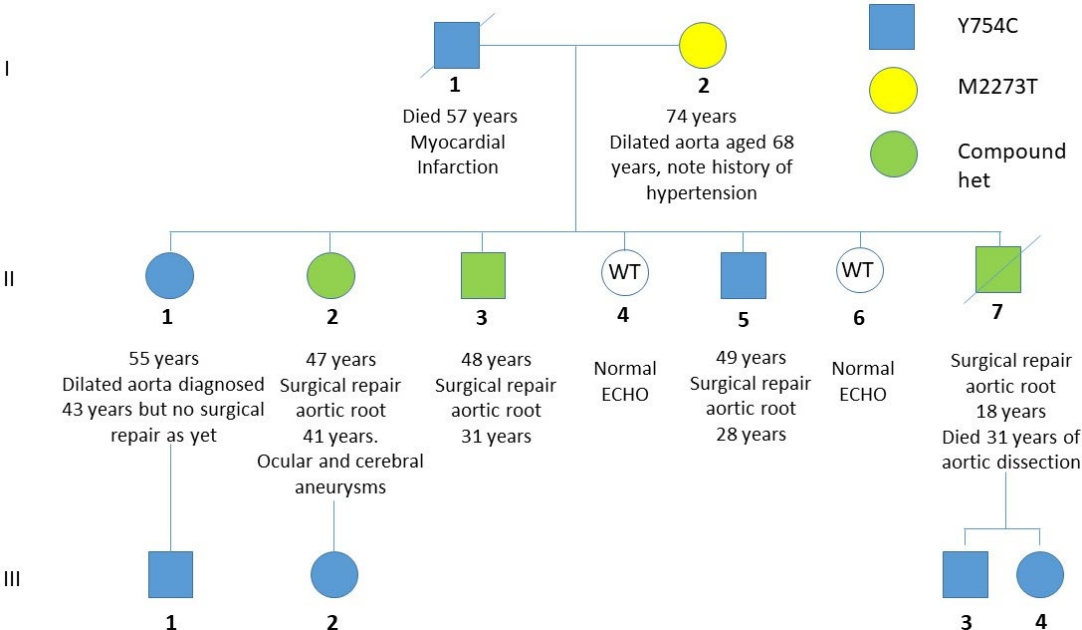
Pedigree showing segregation of both *FBN1* variants

The pedigree is presented in Figure 1. The individuals in the present report include a branch of the pedigree (individual III‐4 and his family) presented in the previous study (Summers et al., 2004).

### 3.1 WES

An average of 5.42 Gb of sequence per individual was generated (mean depth of coverage 43‐fold). Two nonsynonymous coding variants in *FBN1* were identified.

A missense variant (NM_000138.5:exon19:c.2261A > G:NP_000129.3:p.Tyr754Cys, rs137854479) was present in all individuals diagnosed with MFS (Figure 1), as previously reported (Summers et al., 2004). It affects a well‐conserved base (genomic evolutionary rate profiling (GERP) score 5.66), and is predicted deleterious by Polyphen (Adzhubei et al., 2010), MutationTaster (Schwarz, Cooper, Schuelke, & Seelow, 2014), and SIFT (Kumar, Henikoff, & Ng, 2009). This replaces a tyrosine with a cysteine in EGF‐like 11 domain (the seventh calcium‐binding EGF‐like domain) (UniProt, 2019), which is likely to impact disulfide bond creation, and compromise fibrillin‐1 protein structure (Schrijver, Liu, Brenn, Furthmayr, & Francke, 1999).

A second nonsynonymous variant in *FBN1* was also identified (NM_000138.5:exon56:c.6818T > C:NP_000129.3:p.Met2273Thr, rs754270535). This variant is rare (MAF = 3.18e‐5 in gnomAD (Karczewski et al. 2019)) and affects a well‐conserved base (GERP 5.91). The altered residue lies in the EGF‐like 39 domain (the 35th calcium‐binding EGF‐like domain) (UniProt, 2019), adjacent to a conserved cysteine, and is predicted damaging by MutationTaster (1.0), tolerated by SIFT (0.2), and benign by Polyphen (0.87). The variant is listed in the UMD‐FBN1 database (exon 55 in this database) (Collod‐Beroud et al., 2003), reported from an individual diagnosed with incomplete MFS (skeletal and cardiac features of MFS but no ocular abnormalities) (Comeglio et al., 2007). The second variant was present in three siblings with MFS, each of whom also carried the known family variant, and in their mother, who has not been diagnosed with MFS.

The three compound heterozygous cases had collectively three children diagnosed with MFS, each carried the previously identified *FBN1* variant (i.e., p.Tyr754Cys) but not the newly identified p.Met2273Thr variant.

### 3.2 Clinical information

Detailed phenotypes are presented in Table 1.

**Table 1:** Clinical features of family members relative to the revised Ghent criteria for Marfan syndrome

Revised Ghent criteria	I‐1	I‐2	II‐1	III‐1	II‐2	III‐2	II‐3	II‐5	II‐7	III‐3	III‐4
FBN1 mutation screening:											
Y754C	+	−	+	+	+	+	+	+	+	+	+
M2273T	−	+	−	−	+	−	+	−	+	−	−
In the presence of a family history:		N/A									
Ectopia lentis AND family history of MFS	+		+	+	+	+	+	+	+	+	+
Systemic score (≥7 points)*AND FH of MFS	NK		–	–	–	–	–	–	+	–	–
Aortic dilatation (Z ≥ 2 above 20 years old, ≥3 below 20 years) + family history of MFS	+		+	+	+	–	+	+	+	–	–
In the absence of a family history:	N/A		N/A	N/A	N/A	N/A	N/A	N/A	N/A	N/A	N/A
Aortic dilatation (Z ≥ 2) AND ectopia lentis		–									
Aortic dilatation (Z ≥ 2) AND *FBN1*		+									
Aortic dilatation (Z ≥ 2) AND Systemic score (≥7 points)*		–									
Ectopia lentis AND FBN1 with known aortic dilatation		–									
*Systemic score	NK										
Wrist AND thumb sign (3) (Wrist OR thumb sign (1))		–	–	–	–	3	3	–	3	–	–
Pectus carinatum deformity (2) (pectus excavatum or chest asymmetry (1))		−	−	2	−	−	−	−	−	−	−
Hindfoot deformity (2) (plain pes planus (1))		−	−	−	−	−	−	1	1	−	−
Pneumothorax (2)		−		−	−	−	2	−	1	−	−
Dural ectasia (2)	NP	NP	NP	NP	NP	NP	NP	NP	NP	NP	NP
Protrusio acetabuli (2)	NP	NP	NP	NP	NP	NP	NP	NP	NP	NP	NP
Reduced US/LS AND increased arm/height AND no severe scoliosis (1)		NP	1	−	1	1	−	−	1	−	−
Scoliosis or thoracolumbar kyphosis (1)		−	1	−	−	−	−	1	−	−	−
Reduced elbow extension (1)		NP	−	−	NP	1	−	−	1	−	−
Facial features (3 of 5) (1) (dolichocephaly, enophthalmos, downslanting palpebral fissures, malar hypoplasia, retrognathia)		−	−	−	1	−	−	−	−	−	−
Skin striae (1)		−	−	−	−	−	−	−	−	−	−
Myopia > 3 diopters (1)	+	−	1	1	1	1	1	1	1	1	1
Mitral valve prolapse (all types) (1)		−	−	−	−	−	−	−	−	−	−
Total score (maximum = 20)	NK	NK	3	3	3	3	6	3	8	1	1
Meets revised Ghent Diagnostic criteria for Marfan Syndrome	Yes	−	Yes	Yes	Yes	Yes	Yes	Yes	Yes	Yes	Yes
Age aortic dilatation first detected	NK	68 years	43 years	29 years (equivocal)	28 years	N/A (normal at 39 years)	20s	NK	16 years	N/A (normal 11 years)	N/A (normal 8 years)
Age of aortic dissection or reparative surgery	N/A (died at 57 years of MI)	N/A	N/A	N/A	41 years	N/A	31 years	AVR aged 28 years; no aortic repair otherwise	18 years	N/A	N/A

Abbreviations: AVR, aortic valve replacement; FH, family history; LS, lower segment; MFS, Marfan syndrome; MI, myocardial infarction; NK, Not Known; NP, Not Performed; US, upper segment.

The father (I‐1), who carried the common family variant (p.Tyr754Cys), was diagnosed with MFS based on ectopia lentis, dilated aorta (not requiring repair), and a known diagnosis of MFS in his siblings and offspring (Summers et al., 2004). He died aged 57 years of a myocardial infarction.

The mother (I‐2), who carried the second variant (p.Met2273Thr), has not been diagnosed with MFS. She had echocardiography at age 65 years following the diagnosis of hypertension, which showed aortic dilatation (>2SD). She has no ocular signs. Her skeletal phenotype has not been assessed (including no measurement of arm span to height ratio and upper to lower segment ratio). She does not have ectopia lentis. She is otherwise well, aged 74 years.

Of their seven children, five have MFS. Three are compound heterozygous (II‐3, II‐2, and II‐7), with early evidence of aortic dilatation (presenting before age 30 years) requiring aortic root repair (at ages 31 years, 41 years, and 18 years, respectively). II‐2 also had cerebral and ocular vasculature aneurysms and II‐7 died aged 31 years (13 years after aortic root repair) from aortic dilatation and dissection. The remaining two individuals with MFS (II‐1 and II‐5) carry only the common family variant (p.Tyr754Cys). II‐1 had aortic dilatation (4 cm diameter at the sinus of Valsalva, *Z*‐score 2.61) diagnosed at age 43 years, without progression over the next 6 years, and to date, has not required surgery (now aged 55 years). II‐5 had aortic root repair at 28 years; since then, aortic root diameter has remained normal requiring no further intervention (now aged 49 years). The remaining two children were clinically and genetically unaffected.

Three offspring of the three compound heterozygous individuals had clinical data and DNA available. All three have been diagnosed with MFS. Each carried the common family variant (p.Tyr754Cys). They had normal echocardiograms when last assessed (at ages 39 years, 11 years, and 8 years).

## 4 DISCUSSION

We present three siblings with compound heterozygous *FBN1* mutations, whose vascular phenotype may be more severe than other members of the pedigree with a single mutation.

All individuals in this family with diagnosed MFS (all of whom have the p.Tyr754Cys variant) have ectopia lentis, though the skeletal phenotype is variable, and not all have aortic dilatation. The three compound heterozygous individuals presented with aortic dilatation at an early age and required surgical intervention; one died at 31 years of aortic dissection and another had extensive aneurysmic disease, including an ocular aneurysm, which has not been reported previously in MFS. One of their singly heterozygous siblings also required aortic root repair at a young age (28 years); the other was diagnosed with aortic dilatation aged 43 years but has not required repair, and the three singly heterozygous offspring do not have aortic root dilatation (noting that two of them are still very young). Additionally, their singly heterozygous father did not require surgical intervention. Their mother carries a variant previously associated with incomplete MFS; however, as far as has been assessed, her phenotype does not meet the revised Ghent criteria for MFS and appears milder than that previously reported (Comeglio et al., 2007). She did not have an echocardiogram prior to developing hypertension. About 4.5% of women with otherwise undifferentiated hypertension have aortic dilatation (Covella et al. 2014); thus, the significance of her aortic root dilatation is unclear.

Compound heterozygosity or homozygosity for *FBN1* mutations in MFS has been reported previously, and a review of all 20 reported individuals from 16 families has been published (Arnaud et al., 2017). The phenotype of these published cases appears more severe than that of their heterozygous parents and/or other family members. Relevantly, varying expression of the wild‐type *FBN1* allele in heterozygous individuals may modify phenotypic severity. Lower expression of wild‐type *FBN1* mRNA was associated with a more severe phenotype (increased risk of ectopia lentis, pectus deformities, and aortic dilatation) with variability in expression postulated from trans‐acting regulators (Aubart et al., 2015). Individuals with a more severe MFS phenotype may have a second variant (pathogenic or of uncertain significance) in another MFS/thoracic aortic aneurysm/cerebral aneurysm gene, for example, *COL4A1*, as demonstrated in nine of 51 severely affected cases (Aubart et al., 2018). The limited number of family members in our pedigree precludes more definitive conclusions regarding possible additive effects of the second variant; and unfortunately, we do not have access to fibroblasts to assess *FBN1* expression.

Of note, neither of the mutations reported here are loss‐of‐cysteine or premature termination codon (PTC) variants, which are associated with a more severe phenotype (Faivre et al., 2007). None of the homozygous cases reported to date have loss‐of‐cysteine or PTC variants, while all of the five reported compound heterozygous cases carried either a loss‐of‐cysteine or PTC variant in one allele had a milder (non‐PTC or non‐loss‐of‐cysteine) missense mutation in trans (Arnaud et al., 2017).

In conclusion, we report here three individuals with MFS and compound heterozygous mutations in *FBN1*, all of whom had severe aneurysmic disease, suggesting a gene dose effect of *FBN1* mutations may contribute to a more severe phenotype.

## CONFLICT OF INTEREST

The authors declare no potential conflict of interest.

## Data Availability

The data that support the findings of this study are available from the corresponding author upon reasonable request.

## References

[mgg31116-bib-0001] Adzhubei, I. A. , Schmidt, S. , Peshkin, L. , Ramensky, V. E. , Gerasimova, A. , Bork, P. , … Sunyaev, S. R. (2010). A method and server for predicting damaging missense mutations. Nature Methods, 7(4), 248–249. 10.1038/nmeth0410-248 20354512 PMC2855889

[mgg31116-bib-0002] Arnaud, P. , Hanna, N. , Aubart, M. , Leheup, B. , Dupuis‐Girod, S. , Naudion, S. , … Boileau, C. (2017). Homozygous and compound heterozygous mutations in the FBN1 gene: Unexpected findings in molecular diagnosis of Marfan syndrome. Journal of Medical Genetics, 54(2), 100–103. 10.1136/jmedgenet-2016-103996 27582083

[mgg31116-bib-0003] Aubart, M. , Gazal, S. , Arnaud, P. , Benarroch, L. , Gross, M. S. , Buratti, J. , … Boileau, C. (2018). Association of modifiers and other genetic factors explain Marfan syndrome clinical variability. European Journal of Human Genetics, 26(12), 1759–1772. 10.1038/s41431-018-0164-9 30087447 PMC6244213

[mgg31116-bib-0004] Aubart, M. , Gross, M. S. , Hanna, N. , Zabot, M. T. , Sznajder, M. , Detaint, D. , … Stheneur, C. (2015). The clinical presentation of Marfan syndrome is modulated by expression of wild‐type FBN1 allele. Human Molecular Genetics, 24(10), 2764–2770. 10.1093/hmg/ddv037 25652400

[mgg31116-bib-0005] Baetens, M. , Van Laer, L. , De Leeneer, K. , Hellemans, J. , De Schrijver, J. , Van De Voorde, H. , … Coucke, P. J. (2011). Applying massive parallel sequencing to molecular diagnosis of Marfan and Loeys‐Dietz syndromes. Human Mutation, 32, 1–10. 10.1002/humu.21525 21542060

[mgg31116-bib-0006] Baudhuin, L. M. , Kotzer, K. E. , & Lagerstedt, S. A. (2015). Increased frequency of FBN1 truncating and splicing variants in Marfan syndrome patients with aortic events. Genetics in Medicine, 17(3), 177–187. 10.1038/gim.2014.91 25101912

[mgg31116-bib-0007] Collod‐Beroud, G. , Le Bourdelles, S. , Ades, L. , Ala‐Kokko, L. , Booms, P. , Boxer, M. , … Boileau, C. (2003). Update of the UMD‐FBN1 mutation database and creation of an FBN1 polymorphism database. Human Mutation, 22(3), 199–208. 10.1002/humu.10249 12938084

[mgg31116-bib-0008] Comeglio, P. , Johnson, P. , Arno, G. , Brice, G. , Evans, A. , Aragon‐Martin, J. , … Child, A. (2007). The importance of mutation detection in Marfan syndrome and Marfan‐related disorders: Report of 193 FBN1 mutations. Human Mutation, 28(9), 928 10.1002/humu.9505 17657824

[mgg31116-bib-0009] Covella, M. , Milan, A. , Totaro, S. , Cuspidi, C. , Re, A. , Rabbia, F. , & Veglio, F. (2014). Echocardiographic aortic root dilatation in hypertensive patients: A systematic review and meta‐analysis. Journal of Hypertension, 32(10), 1928–1935; discussion 1935. 10.1097/HJH.0000000000000286 24979304

[mgg31116-bib-0010] Dietz, H. C. , & Pyeritz, R. E. (1995). Mutations in the human gene for fibrillin‐1 (FBN1) in the Marfan syndrome and related disorders. Human Molecular Genetics, 4, 1799–1809. 8541880 10.1093/hmg/4.suppl_1.1799

[mgg31116-bib-0011] Dietz, H. C. , Pyeritz, R. E. , Puffenberger, E. G. , Kendzior, R. J. Jr , Corson, G. M. , Maslen, C. L. , … Cutting, G. R. (1992). Marfan phenotype variability in a family segregating a missense mutation in the epidermal growth factor‐like motif of the fibrillin gene. Journal of Clinical Investigation, 89(5), 1674–1680. 10.1172/JCI115766 1569206 PMC443046

[mgg31116-bib-0012] Faivre, L. , Collod‐Beroud, G. , Loeys, B. L. , Child, A. , Binquet, C. , Gautier, E. , … Boileau, C. (2007). Effect of mutation type and location on clinical outcome in 1,013 probands with Marfan syndrome or related phenotypes and FBN1 mutations: An international study. American Journal of Human Genetics, 81(3), 454–466. 10.1086/520125 17701892 PMC1950837

[mgg31116-bib-0013] Judge, D. P. , & Dietz, H. C. (2005). Marfan's syndrome. Lancet, 366(9501), 1965–1976. 10.1016/S0140-6736(05)67789-6 16325700 PMC1513064

[mgg31116-bib-0014] Karczewski, K. J. , Francioli, L. , Tiao, G. , Cummings, B. B. , Alfoldi, J. , Wang, Q. … MacArthur, D. G. (2019). Variation across 141,456 human exomes and genomes reveals the spectrum of loss‐of‐function intolerance across human protein‐coding genes. bioRxiv, 531210 https://www.biorxiv.org/content/10.1101/531210v3. 10.1101/531210.

[mgg31116-bib-0015] Kumar, P. , Henikoff, S. , & Ng, P. C. (2009). Predicting the effects of coding non‐synonymous variants on protein function using the SIFT algorithm. Nature Protocols, 4(7), 1073–1081. 10.1038/nprot.2009.86 19561590

[mgg31116-bib-0016] Lee, B. , Godfrey, M. , Vitale, E. , Hori, H. , Mattei, M. G. , Sarfarazi, M. , … Hollister, D. W. (1991). Linkage of Marfan syndrome and a phenotypically related disorder to two different fibrillin genes. Nature, 352(6333), 330–334. 10.1038/352330a0 1852206

[mgg31116-bib-0017] Loeys, B. , De Backer, J. , Van Acker, P. , Wettinck, K. , Pals, G. , Nuytinck, L. , … De Paepe, A. (2004). Comprehensive molecular screening of the FBN1 gene favors locus homogeneity of classical Marfan syndrome. Human Mutation, 24(2), 140–146. 10.1002/humu.20070 15241795

[mgg31116-bib-0018] Loeys, B. L. , Dietz, H. C. , Braverman, A. C. , Callewaert, B. L. , De Backer, J. , Devereux, R. B. , … De Paepe, A. M. (2010). The revised Ghent nosology for the Marfan syndrome. Journal of Medical Genetics, 47(7), 476–485. 10.1136/jmg.2009.072785 20591885

[mgg31116-bib-0019] Marfan, A. (1896). Un cas de déformation congénitale des quartre membres, plus prononcée aux extrémitiés, caractérisée par l'allongement des os avec un certain degré d'amincissement [A case of congenital deformation of the four limbs, more pronounced at the extremities, characterized by elongation of the bones with some degree of thinning]. Bulletins Et Memoires De La Société Medicale Des Hôspitaux De Paris, 13, 220–226.

[mgg31116-bib-0020] McInerney‐Leo, A. M. , Harris, J. E. , Gattas, M. , Peach, E. E. , Sinnott, S. , Dudding‐Byth, T. , … Duncan, E. L. (2016). Fryns syndrome associated with recessive mutations in PIGN in two separate families. Human Mutation, 37(7), 695–702. 10.1002/humu.22994 27038415

[mgg31116-bib-0021] Milewicz, D. M. , & Regalado, E. S. (2017). Heritable thoracic aortic disease overview (Vol. 1993–2019). Seattle, WA: University of Washington. 20301299

[mgg31116-bib-0022] Robinson, P. N. , Arteaga‐Solis, E. , Baldock, C. , Collod‐Beroud, G. , Booms, P. , De Paepe, A. , … Godfrey, M. (2006). The molecular genetics of Marfan syndrome and related disorders. Journal of Medical Genetics, 43(10), 769–787. 10.1136/jmg.2005.039669 16571647 PMC2563177

[mgg31116-bib-0023] Rommel, K. , Karck, M. , Haverich, A. , von Kodolitsch, Y. , Rybczynski, M. , Muller, G. , … Arslan‐Kirchner, M. (2005). Identification of 29 novel and nine recurrent fibrillin‐1 (FBN1) mutations and genotype‐phenotype correlations in 76 patients with Marfan syndrome. Human Mutation, 26(6), 529–539. 10.1002/humu.20239 16220557

[mgg31116-bib-0024] Schrijver, I. , Liu, W. , Brenn, T. , Furthmayr, H. , & Francke, U. (1999). Cysteine substitutions in epidermal growth factor‐like domains of fibrillin‐1: Distinct effects on biochemical and clinical phenotypes. American Journal of Human Genetics, 65(4), 1007–1020. 10.1086/302582 10486319 PMC1288233

[mgg31116-bib-0025] Schwarz, J. M. , Cooper, D. N. , Schuelke, M. , & Seelow, D. (2014). MutationTaster2: Mutation prediction for the deep‐sequencing age. Nature Methods, 11(4), 361–362. 10.1038/nmeth.2890 24681721

[mgg31116-bib-0026] Summers, K. M. , Xu, D. , West, J. A. , McGill, J. J. , Galbraith, A. , Whight, C. M. , … West, M. J. (2004). An integrated approach to management of Marfan syndrome caused by an FBN1 exon 18 mutation in an Australian Aboriginal family. Clinical Genetics, 65(1), 66–69. 10.1111/j.2004.00186.x 15032979

[mgg31116-bib-0027] UniProt, C . (2019). UniProt: A worldwide hub of protein knowledge. Nucleic Acids Research, 47(D1), D506–D515. 10.1093/nar/gky1049 30395287 PMC6323992

